# World Health Organization is losing online credibility towards health-sensitive topics: Infodemiological analysis of Facebook users’ reactions

**DOI:** 10.34172/hpp.2022.48

**Published:** 2022-12-31

**Authors:** Alessandro Rovetta

**Affiliations:** R&C Research, Bovezzo (BS), Italy

**Keywords:** Infodemic, Infodemiology, Public health, Social networking, Trust, World Health Organization

## Abstract

**Background:** The scientific infodemic constitutes one of the greatest threats to public health and safety today. The credibility of the main dissemination agencies is an essential tool for adhering to measures to preserve public health.

**Methods:** The study is a longitudinal retrospective conducted on a web platform to investigate netizens’ infodemic attitude towards World Health Organization. Reactions such as "like," "love," "affection," "surprise," "sadness," "anger," and "derision" were collected under World Health Organization (WHO) Facebook posts on climate change (from 2019 to 2022) and vaccines (from 2021 to 2022). Descriptive statistics, linear regression, and correlation methods were implemented to identify possible trends and relationships with the COVID-19 vaccination campaign.

**Results:** These findings showed a worrying increase in derision reactions about climate change-related posts (up to 22% in November 2022, with a quadratically growing trend over time since December 2020). Furthermore, infodemic reactions such as anger and especially derision made up the majority of emotional reactions to vaccine-related posts since 2021 and up to 44% of total reactions in November 2022 (median since July 2021=9%, IQR: 4%-14%). Finally, there is evidence of a correlation between the start of the COVID-19 vaccination campaign and public distrust towards the WHO, even for issues unrelated to vaccines such as climate change.

**Conclusion:** Based on what is known in the literature, these preliminary findings signal that the WHO is losing online public credibility towards extremely relevant issues for global health. Infodemiological interventions in accordance with the recent literature are urgently required.

## Introduction

 An infodemic can be defined as an epidemic of information capable of compromising public safety and health.^1^ The overlap of reliable information (e.g., well-disclosed scientific evidence) and dis-misinformation (e.g., fake news, conspiracy hypotheses, poorly disclosed preliminary evidence) is able to lead people to distrust institutions and assume dangerous behaviors. The success of health crisis management strategies is based on the awareness and acceptance of scientific knowledge by people.^2,3^ For instance, during the COVID-19 pandemic, a widespread global infodemic caused very serious epidemiological damage. Therefore, it is absolutely essential to apply infoveillance approaches in all the most used media, especially social networks.^4^ However, such a dramatic scenario was possible not only thanks to disinformation but also to serious communication by scientists.^3,5^ In this regard, based on what has been discussed above, it is fundamental that the main health institutions have the necessary credibility to guide the population during times of uncertainty. This research paper investigates the public reactions on the official Facebook page of the most influential and recognized health institution in the world, namely, the World Health Organization (WHO). Indeed, social networks are fertile ground for disseminating false and fabricated news since the degree of moderation is insufficient.^6^ For instance, the COVID-19 social infodemic was so vast and generalized as to induce strong doubts in the population, thus compromising the public credibility of scientifically valid sources.^3,7,8^ Ergo, as also reported by Tsao et al, infoveillance approaches on these platforms are critical to maintaining global public health.^8^ In particular, emoji-related reactions have a great utility and reliability in delineating the public’s feelings towards a given issue.^9,10^ Based on the above, the present study adopts sentiment analysis methods through the examination of the emotional reactions of Facebook users. This approach has been successfully implemented in previous infodemiological surveys.^8-12^ Two primary global health topics, such as climate change and vaccines, were examined.^13^ Indeed, climate change has already irreversibly led to the loss of sea ice, melting glaciers and ice sheets, sea level rise, and more intense heat waves. Scientists predict that global temperature increases from human-made greenhouse gases will continue. Therefore, since severe weather damage will also increase and intensify, it is essential to act immediately to prevent the situation from becoming unmanageable.^14^ Alongside this, vaccines are a fundamental tool for containing infectious diseases. Vaccination is essential not only for the lives directly saved but also to avoid overloading health facilities.^3^ In anticipation of current and future epidemic waves, vaccination adherence is and will be one of the crucial epidemiological determinants for maintaining global public health.

## Material and Methods

###  Data collection

 Reactions such as “like,” “love,” “affection,” “surprise,” “sadness,” “anger,” and “derision” were collected under all posts on climate change (from 2019 to 2022) and vaccines (from 2021 to 2022). To this end, the words “climate” and “vaccine” were entered in the appropriate search bar on the WHO’s official Facebook page.

###  Study design

 A longitudinal retrospective study was conducted to investigate netizens’ infodemic attitude towards WHO. The detailed procedure is reported in full via https://osf.io/fnmbx/. However, a brief summary is provided here. Regarding climate change, reactions of derision were considered infodemic, and reactions of derision and anger were considered potentially infodemic. Regarding vaccines, reactions of derision and anger were considered infodemic, and reactions of derision, anger, and sadness were considered potentially infodemic. A quick qualitative analysis of the posts was carried out to ascertain that there were no contents of a deliberately ironic nature. A quantitative analysis was employed to establish the size and statistical surprise of the effects concerning the chosen tests. The objective was to determine any anomalous differences in infodemic reactions during the COVID-19 crisis.

###  Statistical analysis

 The effect size was evaluated considering the best values ​​and their 95% confidence intervals (95% CI), standard errors (SE), or interquartile ranges (IQR), while the surprise was evaluated through the so-called “surprisals” equals to “S-value = - log_2 _(*P* values)” (which compare the surprise of the result to that of S consecutive test when flipping a coin). Joinpoint and ordinary least squares linear regression analyses were used to identify trends or subtrends after verifying the appropriate assumptions of homoskedasticity (S < 2) and distributive normality of the residuals (S < 2).^15^ Statistics Kingdom software was also adopted.^16,17^ Relationships between COVID-19 total vaccinations and infodemic reactions have been explored.^18^

## Results

###  Climate change-related infodemic reactions

**Figure 1:**
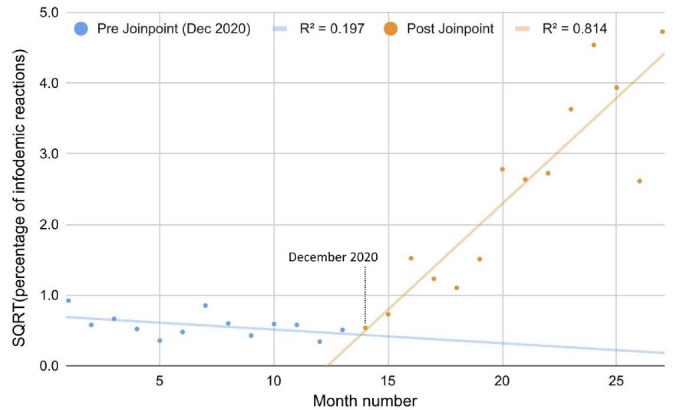
Joinpoint regression analysis of infodemic reactions to WHO climate change posts from 2019 to 2022

 The monthly averages of the infodemic reaction rates relating to climate change posts (y) have grown quadratically from December 2020 (x = 14) onwards: y = (Ax + B)^2^, with A = 0.30 (SE 0.04), S = 17 and B = -3.7 (SE 0.87), S = 10 (R_adj_^2^ = 0.80, 95% CI: [0.48, 0.93] for the linear model shown in Figure 1). The maximum peak of infodemic reactions (i.e., the “laugh” reaction) was reached in November 2022 (over 22%).

###  Vaccine-related infodemic reactions

**Figure 2:**
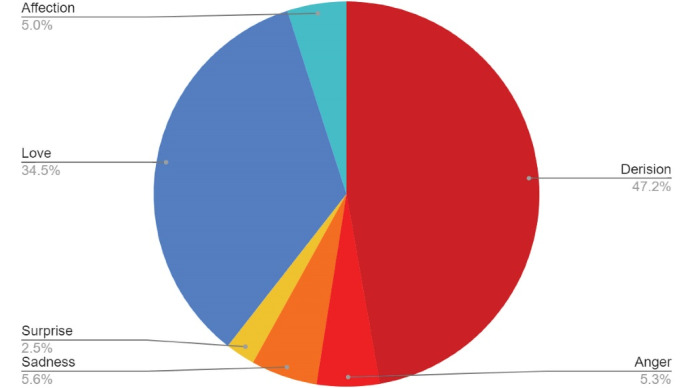
Diagram of emotional reactions to vaccine-related WHO posts

**Figure 3:**
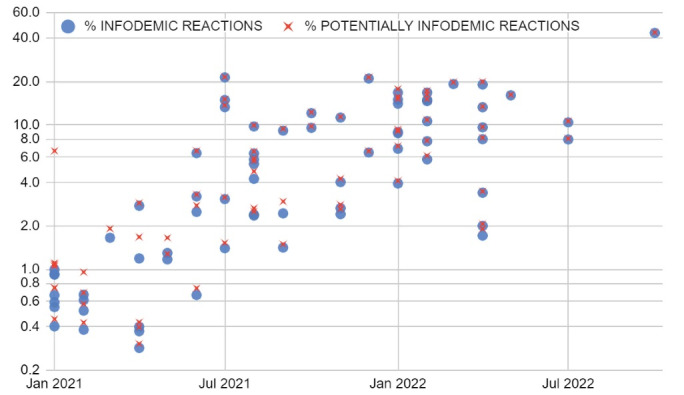
Percentage values ​​of infodemic reactions (derision and anger) and potentially infodemic reactions (derision, anger, and sadness) inherent in vaccine-related WHO posts during 2021 and 2022 (logarithmic scale)

 The infodemic reactions (i.e., derision and anger) made up over 50% of the emotional and total reactions (Figure 2) and ranged from 0.3% to 44% of the total reactions with an overall increase over time (level shifts, Figure 3). The median value from July 2021 onwards was 9%, IQR: [4%; 14%]. In both topics, no substantial drops in total interactions were noted.

###  Relationship between climate change infodemic reactions and COVID-19 vaccines

 The start of the trend reported in Figure 1 regarding infodemic reactions to climate change posts (z) coincided with the beginning of the anti-COVID-19 vaccination campaign (t, expressed in billions). The ordinary least squares linear regression “z = Ct + D,” with C = 0.25 (SE 0.03), S = 17, and D = 0.66 (SE 0.29), S = 4.6 (R_adj_^2^ = 0.81, 95% CI [0.50, 0.94]), showed a marked and surprising correlation.

## Discussion

###  Principal results

 These findings show a marked increase in users’ infodemic reactions to WHO’s Facebook posts. In particular, derision constitutes most of the emotional responses and a substantial fraction of the total reactions regarding climate change and vaccines. There is strong statistical evidence of a substantial and surprising increasing trend in distrust towards WHO-reported information on climate change. Furthermore, although very variable, from July 2021 onwards, the skepticism about the information reported by the WHO on vaccines – especially those against COVID-19 – has been extremely elevated. The highest infodemic peak was reached in a sponsored post in November 2022, where scorn and anger accounted for the dramatic 44% of total reactions. This outlier could indicate a level shift time series. Finally, there is evidence of a correlation between the start of the COVID-19 vaccination campaign and public distrust towards the WHO, even for issues unrelated to vaccines such as climate change.

###  Comparison with the literature and practical implications

 This situation is hazardous and unacceptable. Indeed, these analyses not only denounce the current problematic situation but provide evidence for a further decline in the future. The dramatic increase in infodemic reactions in December 2020 is plausibly due to the widespread dis-misinformation campaign surrounding COVID-19 vaccines. Alongside the – scientifically unjustified – fear of too rapid development, there are determinants related to communication errors, psychology of conspiracies, and the profit of professional disinformers.^3,19,20^ Ergo, it is essential that the scientific community immediately concentrate its efforts on mitigating the infodemiological damage and, according to WHO guidelines, set up projects for creating resilience towards infodemics.^1,3^ Since trust is crucial for encouraging compliance, the first step is to restore and renew the public trust in primary health institutions.^21^ This aspect must not only focus on educating the lay public but also on improving communication, transparency, and scientific credibility of the principal dissemination agencies. Secondly, since young minds are more receptive to learning new content, it is necessary to set up school training programs to prevent similar situations from happening again.^22^ Based on this, the following actions are recommended: A1. Government institutions should establish mandatory communication training programs for all scientists with a disclosure-related role. A2. Educational institutions should create school programs, from kindergarten to university, to train students’ scientific-critical ability, allowing them to manage the excess flow of news by selecting and giving importance to information taken from reliable sources only. A3. Governmental institutions should attempt to educate the lay public through mass media about the difference between reliable and unreliable sources (for example, this could be done with appropriate publicity prior to high-rated television programs). A4. Health institutions should set up departments for infoveillance in order to monitor the health-sensitive information circulating in the population and be able to take eventual countermeasures promptly. A5. Governmental institutions should establish severe penalties for those who knowingly disseminate health disinformation.

 This paper has limitations to discuss. In particular, the sentiment analysis based on emoticons did not allow for determining precisely why the user chose a specific reaction. Furthermore, detecting users’ error rate (misclicks) in attributing the desired reaction was impossible. Secondly, these findings alone cannot demonstrate causality but are relevant in light of the literature already published on the topic (through the hypothesis targeting process).^23^ Finally, the conclusions drawn are valid only for netizens registered on the Facebook platform interacting with the WHO’s official page.

## Conclusion

 Based on what is known in the literature, these preliminary evidence suggests that WHO is losing online public credibility towards sensitive and extremely relevant issues for global health and the future of humanity, such as vaccines and climate change. Infodemiological interventions are urgently required to improve communication techniques and educate the lay public and scientific community regarding the concept of scientific reliability.

## Acknowledgements

 I thank Lucia Castaldo for her support and the infodemiological contribution.

## Funding

 This research did not receive funding.

## Data Availability

 All data is reported in full in the manuscript, supplemental material, or links provided in the references.

## Ethical Approval

 Not applicable.

## Competing Interests

 The author declares that he has no conflicts of interest.
